# Association of Clonal Hematopoiesis With Incident, Late‐Onset, Seropositive Rheumatoid Arthritis

**DOI:** 10.1002/art.70133

**Published:** 2026-04-16

**Authors:** Kun Zhao, Yash Pershad, J. Brett Heimlich, Michelle Ormseth, C. Michael Stein, Brian Sharber, Caitlyn Vlasschaert, Alexander G. Bick, Robert W. Corty

**Affiliations:** ^1^ Division of Genetic Medicine and Clinical Pharmacology, Department of Medicine Vanderbilt University Medical Center Nashville Tennessee; ^2^ Division of Cardiovascular Medicine, Department of Medicine Vanderbilt University Medical Center Nashville Tennessee; ^3^ Division of Rheumatology, Department of Medicine Vanderbilt University Medical Center Nashville Tennessee; ^4^ Department of Medicine Queen's University Kingston Ontario Canada

## Abstract

**Objective:**

Clonal hematopoiesis (CH), defined by acquired driver mutations in hematopoietic stem cells, is associated with many inflammatory diseases of aging. We investigated whether CH and its subtypes, CH of indeterminate potential (CHIP) and mosaic chromosomal alteration (mCA), are associated with incident rheumatoid arthritis (RA) and whether complement modifies these associations.

**Methods:**

CHIP was detected in NIH All of Us, Vanderbilt BioVU, and UK Biobank; mCA was detected in UK Biobank. A harmonized, high‐specificity phenotyping algorithm was applied across biobanks to identify participants with seropositive and seronegative RA (SPRA and SNRA). Age‐scale survival models assessed the effect of CH on risk of incident RA. Effect modification was tested with interaction models with genetically predicted complement protein levels.

**Results:**

Among 612,989 participants, 30,840 had CHIP, 1,535 had incident SPRA, and 1,090 had incident SNRA. CHIP was associated with an increased risk of incident SPRA (pooled hazard ratio [HR] 1.26; confidence interval [CI] 1.03–1.52; *P* = 2.3 × 10^−2^), driven primarily by *DNMT3A*‐mutated CHIP and late‐onset RA (HR 1.45; CI 1.13–1.96; *P* = 3.6 × 10^−3^) but not SNRA. Autosomal mCA and mosaic loss of chromosome Y (mLOY) were associated with an increased risk of incident SPRA (HR 2.12 and 2.85; CI 1.18–3.8 and 1.57–5.19; *P* = 1.2 × 10^−2^ and 6.1 × 10^−4^) but not SNRA. Higher genetically predicted levels of C1r and C1s attenuated the CHIP–SPRA association.

**Conclusion:**

Age‐related CH, including *DNMT3A*‐CHIP, autosomal mCA, and mLOY, are risk factors for incident SPRA but not SNRA, supporting a genotype‐ and serostatus‐specific link between somatic mutation and RA, with the classical complement pathway as a potential modifier.

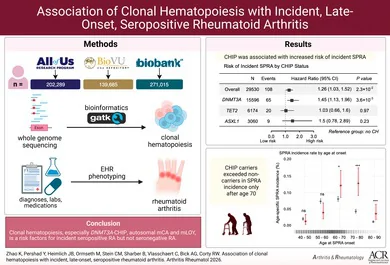

## INTRODUCTION

Rheumatoid arthritis (RA) is a systemic, autoimmune disease characterized by chronic inflammation and heterogeneous clinical trajectories across age, serologic status, and treatment response.^1^ RA primarily affects older adults, though people of all ages can be affected.^2^ RA with onset at the age of 60 years or older has been termed late‐onset RA (LORA).^3^ LORA has a distinct genetic profile, higher levels of inflammatory cytokines, and inferior response to therapy.^4^, ^5^, ^6^, ^7^ The mechanisms driving LORA remain incompletely understood. There is a pressing need for advancements in the diagnosis and care of LORA as its incidence is increasing over time.^2^ The pathogenesis of seropositive RA (SPRA; defined by presence of rheumatoid factor [RF] or anti–cyclic citrullinated peptide antibodies) involves antigen‐driven clonal selection on lymphocytes.^8^ But the role of clonal selection among hematopoietic stem cells, a common phenomenon in older adults, remains much less explored.

The two most common forms of clonal hematopoiesis (CH) are CH of indeterminate potential (CHIP) and mosaic chromosomal alteration (mCA). CHIP is defined by the clonal expansion of hematopoietic stem cells carrying a small somatic mutation in a leukemogenic gene.^9^, ^10^, ^11^ Seventy‐five percent of CHIP cases are driven by mutation in one of three epigenetic regulators, *DNMT3A*, *TET2*, and *ASXL1*.^12^ CHIP is rarely observed among people in their 40s and younger (<1%) but is present in ~15% of individuals in their 70s.^12^ Although typically asymptomatic, CHIP skews hematopoiesis toward myelopoiesis, causes chronic inflammation, and confers elevated risks for myeloid neoplasms, cardiovascular disease, giant cell arteritis, gout, and all‐cause mortality.^10^, ^13^, ^14^, ^15^ mCA, the other form of CH, is defined by large‐scale chromosomal gain, loss, or copy‐neutral loss of heterozygosity of the autosomes or sex chromosomes (ie, mosaic loss of chromosome X, known as mLOX, and mosaic loss of chromosome Y, known as mLOY). mCA is also common among older adults, can be asymptomatic, and confers risk for a distinct, but less well understood, set of diseases of aging.^16^, ^17^

Recent investigations have begun to elucidate the relationship between CH and RA. Hiitola et al found that CHIP is associated with prevalent RA in a genotype‐ and seropositivity‐specific manner, noting that the rate of *DNMT3A* CHIP is elevated among patients with SPRA and the rate of *TET2* CHIP is elevated among patients with seronegative RA (SNRA).^18^ But disease ascertainment was based solely on diagnosis codes, an approach that has been found to have specificity of around 55%.^19^, ^20^, ^21^ Similarly, Uchiyama et al highlighted the critical importance of age at disease onset, reporting that male participants with LORA are enriched for mLOY compared to age‐matched controls but participants with young‐onset RA are paradoxically depleted for mLOY compared to matched controls.^22^ However, both studies used only cross‐sectional data, limiting insights into the time course of the relationship. Furthermore, studies to date have offered limited insight into the mechanistic link between CH, which heightens innate immune activation, and RA, which has prominent adaptive immune features. The complement system functionally links the innate and adaptive immune system.^23^ Notably, C3d‐tagged antigens stimulate B cell activation ~10,000 times more efficiently than nontagged antigens.^23^ Thus, we hypothesize that the complement pathway may influence the association between CH and RA.

To address these questions, we performed a meta‐analysis across three large biobanks: the NIH All of Us Research Program (AoU), Vanderbilt's BioVU, and the UK Biobank (UKB). We leveraged extensive electronic health records (EHR) and genetic sequencing data to study over 600,000 research participants. We employed established bioinformatic pipelines for CH detection, a rigorous RA phenotyping strategy, and longitudinal study design to (1) identify high‐confidence cases of SPRA and SNRA, (2) comprehensively characterize the effects of CHIP and mCA on risk of incident SPRA and SNRA, (3) dissect the effects of CHIP on risk for cardiovascular disease and death among research participants with RA, and (4) test for effect modification by genetically predicted complement protein levels.

## METHODS

### Study cohorts

De‐identified data were obtained from three biobanks with linked EHR and either whole exome or whole genome sequencing (WES/WGS): AoU, BioVU, and UKB. AoU is a population sample in which participants provide a blood sample at a time of their choosing, whereas BioVU is a hospital‐based cohort, enriched for participants with chronic disease, in which blood samples left over after clinical testing were used for sequencing, and UKB is a prospectively designed study in which participants were invited to the assessment center on a medically arbitrary date.

### Inclusion, exclusion, and case ascertainment

Inclusion and exclusion criteria and RA disease ascertainment criteria were applied consistently across the biobanks.

#### Inclusion criteria

Research participants were included in the study only if they had (1) adequate genetic data for CHIP ascertainment, (2) adequate clinical data for RA disease ascertainment (two or more diagnosis codes spanning a year or more, one or more laboratory value, and one or more medication prescription), and (3) adequate engagement with the health care system that RA would likely be diagnosed if it were present (ie, five or more total diagnosis codes).

#### Exclusion criteria

Research participants were excluded if they were aged <40 years at the time of the blood sample used for DNA sequencing because CHIP is very rare among the young.^11^, ^24^ Similarly, research participants were excluded if they had prevalent hematologic cancer (defined by diagnosis codes in Supplementary Table 1).^9^ As smoking is a risk factor for both CH and RA, smoking status was a critical covariate. It was inferred from questionnaires administered to all participants in AoU, BioVU, and UKB, and participants who did not answer were excluded.

#### Follow‐up

Health records in AoU and BioVU are updated approximately annually to include all available data; thus, follow‐up is until last available diagnosis code. In UKB, ambulatory records are kept by general practitioners and are available for approximately 45% of the cohort with a truncation date in 2016 as described in the UKB data showcase, defining the censoring date for participants with no diagnosis of RA. Only participants with available primary care data were included in the study (Figure 1).

**Figure 1:**
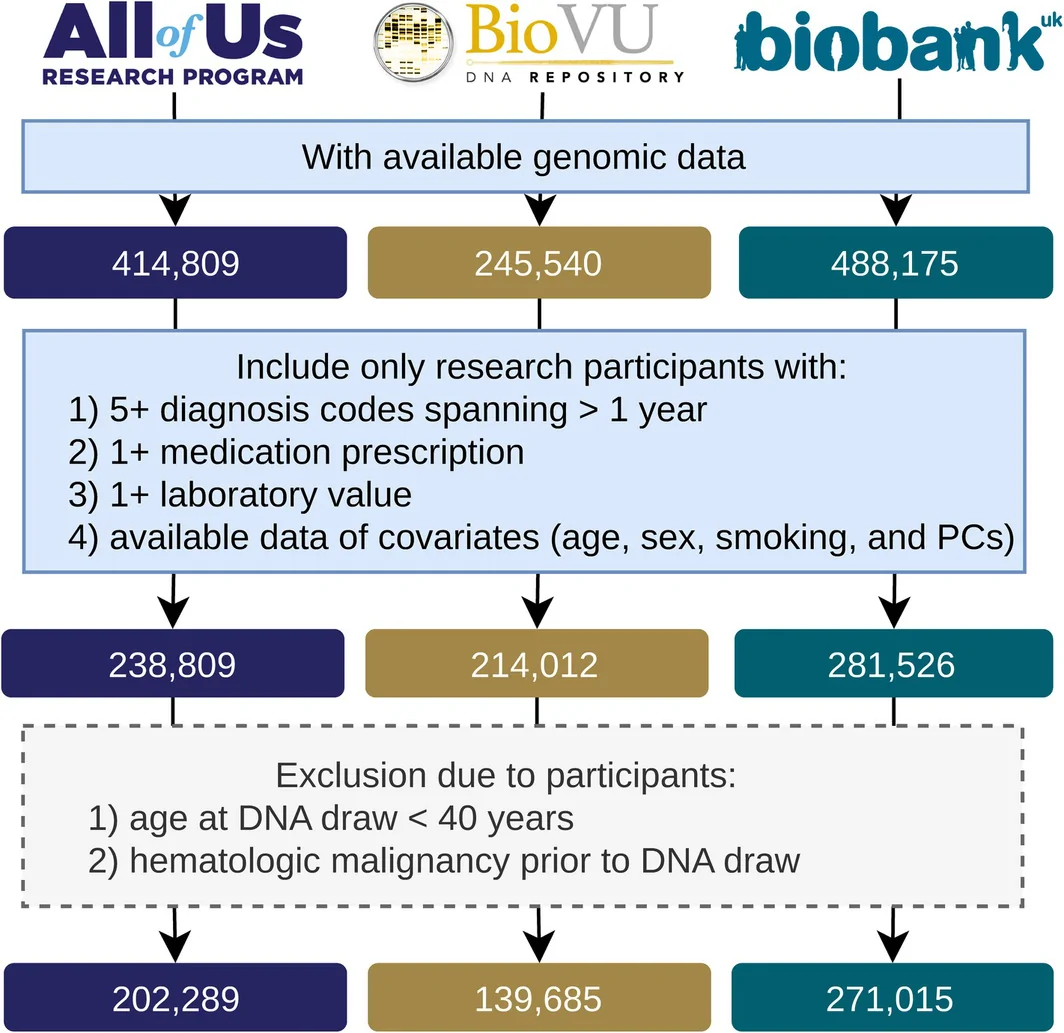
Flow diagram summarizing inclusion and exclusion in the All of Us Research Program, Vanderbilt BioVU, and UK Biobank. PC, principle component.

### RA ascertainment

We used a combination of diagnosis codes, laboratory data, and medication prescription to identify research participants with RA. This approach has been found to have sensitivity and specificity of approximately 85% and 99%, respectively.^19^, ^25^, ^26^, ^27^

#### Diagnosis codes

For BioVU, in which diagnoses are recorded by *International Classification of Diseases, Ninth Revision* (ICD‐9) and ICD‐10 codes, codes matching “M05” were considered SPRA, codes matching “M06” were considered SNRA (excluding “M06.1” and “M06.4”), and codes matching “714.0” were considered RA not otherwise specified. For AoU and UKB, in which diagnoses are recorded in the Systematized Nomenclature of Medicine vocabulary, relevant codes were identified with a search strategy described in the Supplementary Appendix that resulted in a code list provided in Supplementary Table 2.

#### Medications

The list of disease‐modifying antirheumatic drugs (DMARDs) was defined as the combination of conventional synthetic DMARDs, biologic DMARDs, and targeted synthetic DMARDs (Supplementary Table 3).

#### Laboratory values

We identified concepts that capture RF and anti–cyclic citrullinated peptide (anti‐CCP) and developed a careful parsing method to assign each result as either positive or negative, as detailed in the Supplementary Appendix. Each participant was considered seropositive by laboratory values if they had a positive RF or anti‐CCP results, negative if they had a result for at least one with no positive results, and not applicable otherwise.

#### Date of disease onset

Participants with RA were considered to have developed the disease on the date the diagnosis code first appeared in their medical records. Cases were considered as “prevalent” when diagnosed on or before the date of the blood sample used for DNA sequencing and “incident” when diagnosed after the blood sample.

#### Osteoarthritis controls

Controls with osteoarthritis were identified in BioVU as participants with phecode MS_708 and no instances of M05 or M06.

### CHIP detection

Based on genetic sequencing data availability, CHIP calling was performed with WGS for AoU (depth ~40×) and BioVU (depth ~35×) and with WES for UKB (depth ~40×). For each cohort, the same CHIP‐defining somatic single nucleotide variants and short indels were identified per established variant detection methods as we have previously described.^28^, ^29^ Briefly, 74 genes known to harbor CHIP‐driving variants were screened for somatic variants using the *Mutect2* to identify putative CHIP variants (Supplementary Table 4).^10^, ^30^, ^31^ The variant allele fraction of the CHIP clone was visualized with violin plots.

### mCA detection

mCA detection in UKB was previously described. In brief, mCAs in blood DNA genotyping intensity data were detected using a validated statistical phasing‐based approach.^32^

### Genetically predicted complement protein levels

To investigate the mechanistic role of the classical complement pathway without the confounding effects of active inflammation or reverse causation, we used genetically predicted protein levels as a proxy for stable, lifelong systemic exposure. Briefly, we retrieved genetic effect estimates from the INTERVAL study and summed the genotypes of participants of European ancestry weighted by those effects as detailed in the Supplementary Appendix.^33^

### Genetic principal components

Genetic principal components were retrieved from each biobank's central data repository, in which they have been deposited for wide dissemination after applying standard bioinformatic tools to variant tables as detailed in the Supplementary Appendix.^34^, ^35^, ^36^

### Survival models for incident RA

To estimate the CHIP‐associated risk of incident RA, Cox proportional hazards models were used. Because the three biobanks obtained the blood draw used to define CHIP status at times with different health contexts and empirically at a wide range of ages, implying a wide range of baseline risk for age‐associated conditions such as RA, two time scales were evaluated: the “time‐since‐blood‐draw” scale and the “age” scale.^37^, ^38^ Using the Grambsch–Therneau test, the proportional hazards assumption was violated in BioVU and AoU on the “time‐since‐blood‐draw” scale, but on the “age” scale, no proportional hazards violations were observed (Supplementary Table 7).^39^ Therefore, all time‐to‐event analyses were conducted on the age scale. Using the age scale, each participant entered the risk pool at the age at which the blood sample was drawn and exited the risk pool at the age they were diagnosed with RA (event) or the end of the available follow‐up data (censoring). All time‐to‐event analyses included covariates of biologic sex, smoking status, and genetic principal components (PC1–PC5). Age‐scale time‐to‐event analysis accounts for the effect of age comprehensively and nonparametrically, so no parametric correction for age effects on risk for RA was appropriate.^37^ The model described here was applied for both SPRA and SNRA, first estimating the effect of all CHIP and then CHIP driven by each of the three most common drivers (*DNMT3A*, *TET2*, and *ASXL1*). Estimated hazard ratios and their confidence intervals (CIs) were visualized with forest plots. A nonparametric time‐to‐event modeling approach was considered but, due to the critical need for covariate adjustment, was not selected for analysis.

### Logistic regression models for prevalent RA

To estimate the CHIP‐associated odds of prevalent RA, logistic regression was used. Covariates were biologic sex, smoking status, genetic principal components (PC1–PC5), and participant's age at the time of blood draw. As described in the previous paragraph, the model described here was applied to both SPRA and SNRA, first estimating the effect of all CHIP and then CHIP driven by each of the three most common drivers. Estimated odds ratios (ORs) and their CIs were visualized with forest plots.

### Survival models for all‐cause mortality

Among participants with RA, the impact of CHIP on all‐cause mortality was assessed using age‐scale Cox proportional hazards models, adjusting sex, smoking status, and genetic principal components (PC1–PC5), as mentioned in the previous paragraph. Kaplan–Meier curves were generated to visually compare survival across CHIP and non‐CHIP groups, and differences were assessed using the log‐rank test. Empirical survival fraction over time was visualized with Kaplan–Meier plots, and estimated hazard ratios and their CIs were visualized with forest plots.

### Meta‐analysis combining results from three biobanks

Inverse variance‐weighted meta‐analysis is a statistical method that can improve the precision of effect estimates by combining information across studies. Specifically, it weights the effect estimated in each study proportional to the inverse of its variance.^40^ The across‐studies degree of heterogeneity (I^2^ statistic) was used to determine whether the random or fixed effect model was used: in cases in which I^2^ > 50%, the random effect model was used; otherwise the fixed effects model was used, consistent with generally accepted practice.^40^ Meta‐analyses were visualized with forest plots.

### Effect modification by genetically predicted complement levels

We tested for effect modification by modifying the age‐scale survival analysis described earlier in this section to include an interaction term between CHIP status and genetically predicted complement levels.

### Multiple hypothesis testing corrections

Multiple hypothesis testing correction was performed using the Bonferroni method when five or fewer hypotheses were tested, as in the analyses of how specific CHIP driver genes affect the risk or odds of RA and risk of death, and the Benjamini–Hochberg method when six or more hypotheses were tested, as in the analyses of effect modification by predicted complement levels.^41^, ^42^

### Data availability statement

Primary data from the UKB and NIH All Of Us biobanks are public, whereas primary data from BioVU require an institutional account at Vanderbilt University Medical Center. Processed data including CHIP calls, mCA calls, covariates, and phenotype ascertainment will be shared upon request after requester completion of relevant data security and privacy trainings.

## RESULTS

### Sociodemographic characteristics

After applying the unified inclusion and exclusion criteria, a total of 612,989 participants were included across the three biobanks: 202,289 from AoU, 139,685 from BioVU, and 271,015 from UKB (Figure 1). Their sociodemographic characteristics are presented in Table 1. Across cohorts, participants were middle‐aged to older adults at the time of blood sample collection, with median ages ranging from 58.0 to 61.1 years. Women composed over half of each cohort (53.4%–59.6%). Smoking history was common across all three cohorts, with the highest prevalence observed in UKB. AoU and BioVU included more racially diverse participants, whereas UKB was predominantly White.

**Table 1:** Sociodemographic characteristics of the three cohorts studied, separately and in aggregate*

	NIH All of Us (n = 202,289)	BioVU (n = 139,685)	UK Biobank (n = 271,015)	Total (N = 612,989)
Age at blood sample, median (IQR), y	61.1 (52.2–69.5)	58.9 (50.3–68.0)	58.0 (50.0–63.0)	58.0 (50.8–66.3)
Women, n (%)	120,600 (59.6)	75,085 (53.4)	150,584 (55.6)	346,269 (56.5)
Ever smoked, n (%)	87,733 (43.4)	50,459 (36.1)	121,336 (44.8)	259,528 (42.3)
Race, n (%)				
White	121,558 (60.1)	116,082 (83.0)	260,119 (96.0)	497,759 (81.2)
Black or AA	33,775 (16.7)	14,713 (10.5)	3,769 (1.4)	52,257 (8.5)
East Asian	3,971 (2.0)	1,761 (1.3)	7,127 (2.6)	12,859 (2.1)
With clonal hematopoiesis, n (%)				
Overall CHIP	10,799 (5.4)	11,321 (8.1)	8,720 (3.2)	30,840 (5.0)
DNMT3A	5,582 (2.8)	5,399 (3.9)	5,390 (2.0)	16,371 (2.7)
TET2	2,181 (1.1)	2,989 (2.1)	1,313 (0.5)	6,483 (1.1)
ASXL1	888 (0.4)	1,319 (0.9)	1,022 (0.4)	3,229 (0.5)
With prevalent SPRA or SNRA, n (%)				
SPRA	1,523 (0.75)	1,758 (1.26)	689 (0.25)	3,970 (0.65)
SNRA	862 (0.43)	1,286 (0.92)	394 (0.15)	2,542 (0.41)
With incident SPRA or SNRA, n (%)				
SPRA	217 (0.10)	1,101 (0.79)	217 (0.08)	1,535 (0.25)
SNRA	139 (0.07)	798 (0.57)	153 (0.06)	1,090 (0.18)
Years of follow‐up, median (IQR)	2.1 (0.5–4.0)	4.3 (1.7–10.3)	12.3 (11.6–13.1)	8.6 (2.3–12.3)

* AA, African American; CHIP, clonal hematopoiesis of indeterminate potential; IQR, interquartile range; SNRA, seronegative rheumatoid arthritis; SPRA, seropositive rheumatoid arthritis.

### CHIP

Across the three cohorts, a total of 30,840 participants (5.0%) had one or more detectable CHIP mutation(s), with rates of 5.4% in AoU, 8.1% in BioVU, and 3.2% in UKB (*P* < 0.001 for differences across cohorts). CHIP carriers were, on average, older than noncarriers (mean age 65.8 vs 58.4 years, *P* < 0.001). Most CHIP clones were driven by mutations in *DNMT3A* (48%), *TET2* (21%), and *ASXL1* (10%), which together accounted for 79% of all detected driver mutations (Supplementary Figure S1). The aggregate meta‐cohort and each individual cohort demonstrated an increase in the rate of CHIP with age, but the absolute frequency differed across biobanks, with BioVU showing the highest prevalence at all age groups (Supplementary Figures S2A and S2B).

### Prevalent and incident RA

SPRA and SNRA were identified in 3,970 (0.65%) and 2,542 (0.41%) of the combined cohort, respectively. Prevalent SPRA (first diagnosed before the blood draw in which CHIP status was ascertained) occurred in 1,523 (0.75%), 1,758 (1.26%), and 689 (0.25%) participants in AoU, BioVU, and UKB, respectively. Prevalent SNRA occurred in 862 (0.43%), 1,286 (0.92%), and 394 (0.15%) participants in AoU, BioVU, and UKB, respectively. Incident SPRA (first diagnosed after the blood draw in which CHIP status was ascertained) occurred in 217 (0.10%), 1,101 (0.79%), and 217 (0.08%) participants in AoU, BioVU, and UKB, respectively, whereas incident SNRA occurred in 139 (0.07%), 798 (0.57%), and 153 (0.06%) participants in AoU, BioVU, and UKB, respectively.

### CHIP is associated with increased risk of incident SPRA but not SNRA

In a three‐cohort meta‐analysis, a subtype‐specific relationship between CHIP and risk of incident RA was observed, with a clear distinction between SPRA and SNRA. CHIP was associated with increased risk of incident SPRA (pooled hazard ratio [HR] 1.26; 95% CI 1.03–1.52; *P* = 2.3 × 10^−2^; I^2^ = 14%; Figure 2A). Gene‐stratified analyses revealed that this association was primarily driven by *DNMT3A*‐CHIP (HR 1.45; 95% CI 1.13–1.96; *P* = 3.6 × 10^−3^). This association was considered statistically significant because it met the corrected *P* value of 0.05 divided by 3 assigned by the Bonferroni method due to the three genes tested. By contrast, *TET2*‐CHIP was not associated with incident SPRA (Figure 2A, Supplementary Figure S3) nor was *ASXL1*‐CHIP, though effect heterogeneity limited their interpretability (Figure 2A, Supplementary Figure S3). Neither overall CHIP nor any specific CHIP driver gene was associated with incident SNRA (Figure 2B, Supplementary Figure S4). The distribution of CHIP driver mutations and their variant allele fractions was similar between individuals who developed SPRA and those who developed SNRA (Supplementary Figure S5A–D).

**Figure 2:**
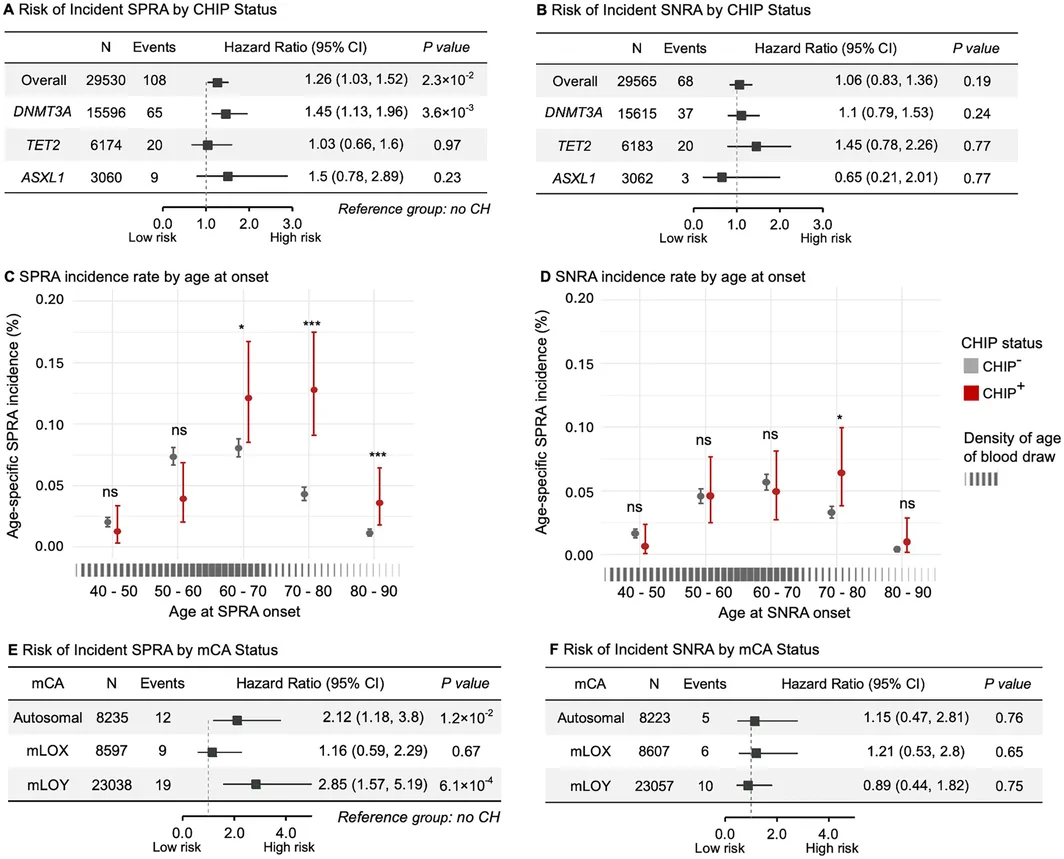
Association of CH with incident SPRA and SNRA. Forest plots show three‐biobank meta‐analytic effects of CHIP and subtypes on risk of incident (A) SPRA and (B) SNRA after correcting for biologic sex, smoking status, and five genetic principal components. “N” indicates the number of CH carriers and “Events” indicates the number of incident SPRA/SNRA cases among CH carriers. Incidence of (C) SPRA and (D) SNRA was calculated by decade and compared across CHIP status. Gray bars along the bottom of panels C and D indicate relative frequency of the blood draws from which CHIP status was inferred, where darker areas indicate the ages when more participants had blood draws performed. Forest plots show the mCA‐associated risk of incident (E) SPRA and (F) SNRA after correcting for biologic sex, smoking status, and five genetic principal components in UKB. *P < 0.05, ***P < 0.0005. CH, clonal hematopoiesis; CHIP, clonal hematopoiesis of indeterminate potential; CI, confidence interval; mCA, mosaic chromosomal alteration; mLOX, mosaic loss of chromosome X; mLOY, mosaic loss of chromosome Y; ns, not significant; SNRA, seronegative rheumatoid arthritis; SPRA, seropositive rheumatoid arthritis; UKB, UK Biobank.

### CHIP is associated with late‐onset SPRA but not early‐onset SPRA

The association of CHIP with incident but not prevalent SPRA presented an apparent conflict. We investigated further by examining the incidence of SPRA by decade and comparing it to the age when blood samples were taken for DNA sequencing (Figure 2C, 2D). Most blood samples were taken in middle age, and CHIP carriers exceeded noncarriers in SPRA incidence only in older age. The largest CHIP‐associated difference in SPRA incidence was observed among research participants age 70 to 80 years, an RA subtype that has been termed LORA (*P* < 0.005; Figure 2C).^22^ In contrast, there was no difference in SPRA incidence by CHIP status among people aged <60 years, suggesting that early‐onset RA is largely unrelated to CHIP.

### Autosomal mCAs and mLOY are associated with increased risk of incident SPRA but not SNRA

Survival analyses in UKB (the only biobank in which mCA data are available) revealed striking associations with SPRA but none for SNRA. Autosomal mCA was associated with an increased risk of incident SPRA (HR 2.12; 95% CI 1.18–3.80; *P* = 1.2 × 10^−2^; Figure 2E). Among male participants, mLOY was associated with an increased risk of SPRA (HR 2.85; 95% CI 1.57–5.19; *P* = 6.1 × 10^−4^; Figure 2E). No significant association was observed between mLOX and risk of SPRA among female participants (HR 1.16; 95% CI 0.59–2.29) nor between any class of mCA and incident SNRA (Figures 2E and 2F).

### Systemic inflammation in CHIP ^+^ and CHIP ^−^ SPRA

Given the association between CHIP and incident SPRA, we sought to assess whether participants with CHIP developed a subtype of SPRA with higher levels of systemic inflammation as manifested in the C‐reactive protein (CRP) level. Therefore, we compared the last CRP measurement before diagnosis of incident SPRA between patients with and without CHIP and compared them to controls without CHIP who developed osteoarthritis. SPRA groups exhibited substantially higher CRP levels than osteoarthritis controls (*P* < 0.001). CRP levels were numerically higher in CHIP^+^ SPRA compared with CHIP^−^ SPRA participants (median 14.7 vs 13.1 mg/L; *P* = 0.048; Supplementary Figure S6).

### CH is not associated with prevalent RA

In contrast to the clear associations between CH and incident SPRA, there was no association between CH and prevalent RA, neither SPRA nor SNRA (Supplementary Figure S7A and S7B). In a meta‐analysis across the three cohorts, the odds of having prevalent SPRA did not differ between CHIP carriers and noncarriers, nor was *DNMT3A*‐, *TET2*‐, or *ASXL1*‐CHIP associated with increased SPRA prevalence. Similarly, in UKB, none of autosomal mCA, mLOX, or mLOY was enriched among people with prevalent SPRA. Similar patterns were observed for prevalent SNRA, with no significant associations for overall CHIP, individual driver genes, or any mCA type.

### CHIP and all‐cause mortality among patients with SPRA and SNRA

Differential mortality is of absolute interest and could also explain the apparent conflict between incident and prevalent RA described previously; therefore, we examined the risk of all‐cause mortality among research participants with prevalent SPRA and SNRA with or without CHIP. In the overall study population, CHIP, both overall and by individual driver genes, was associated with increased mortality risk (Supplementary Figure S8). Among individuals with SPRA, CHIP carriers had shorter survival than noncarriers by the log‐rank test (*P* = 0.04; Figure 3A). Age‐scale survival analysis revealed that overall CHIP was not significantly associated with mortality (HR 1.47; 95% CI 0.99–2.19), but *TET2*‐CHIP was associated with an increased risk of mortality (HR 2.23; 95% CI 1.05–4.74; *P* = 3.8 × 10^−2^; Figure 3C). Analysis of other common CHIP drivers and mCA among participants with SPRA found no association with mortality (Figure 3C). In contrast to SPRA, neither CHIP nor any subtype nor any mCA was associated with mortality among participants with SNRA (Figures 3B and 3D).

**Figure 3:**
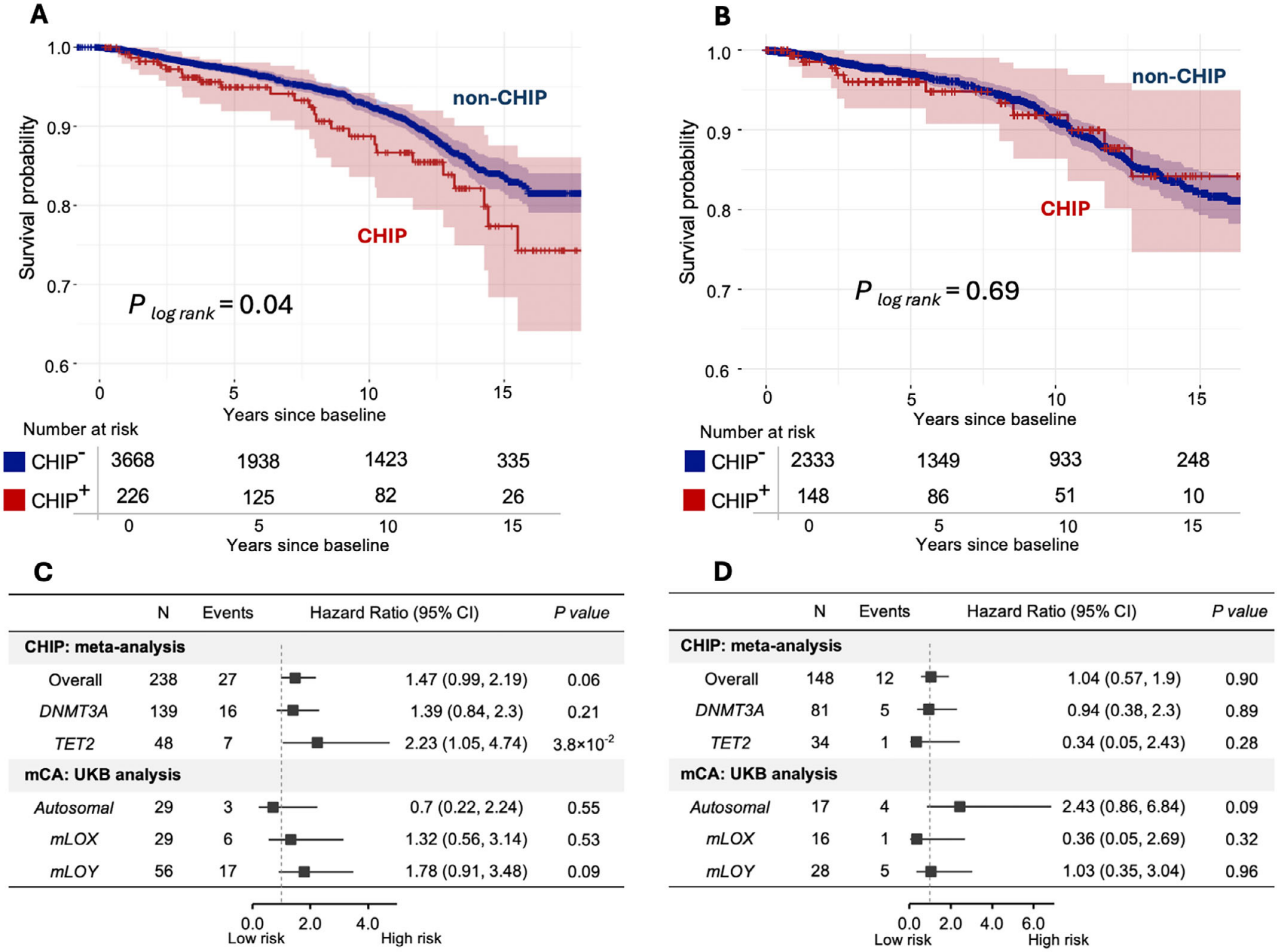
The association between CH and mortality among participants with RA. Kaplan–Meier plots of overall survival for patients with prevalent (A) SPRA and (B) SNRA, stratified by CHIP status. Cox regression was performed to evaluate the association between CH with all‐cause mortality among patients with (C) SPRA and (D) SNRA, correcting for effects of biologic sex, smoking status, and five genetic principal components. CH, clonal hematopoiesis; CHIP, clonal hematopoiesis of indeterminate potential; CI, confidence interval; mCA, mosaic chromosomal alteration; mLOX, mosaic loss of chromosome X; mLOY, mosaic loss of chromosome Y; RA, rheumatoid arthritis; SNRA, seronegative rheumatoid arthritis; SPRA, seropositive rheumatoid arthritis; UKB, UK Biobank.

### Genetically predicted C1s and C1r modify the effect of CHIP on risk of SPRA

To investigate the role of classical complement in modifying the CHIP‐associated risk for SPRA, we performed age‐scale survival analysis with an interactive effect between genetically predicted complement levels and CH status. These analyses revealed effect modification between CHIP and C1s (*P* = 2.2 × 10^−3^, adjusted *P* [*P*_adj_] = 0.013) and C1r (*P* = 1.6 × 10^−2^, *P*_adj_ = 0.049), indicating that higher genetically predicted complement levels attenuate the CHIP‐associated risk of SPRA. In contrast, no significant interaction effect was observed for SPRA and any other complement components (*P* ≥ 0.4; Figure 4). A sensitivity analysis conducted only among people with CHIP examined the effect of genetically predicted complement levels on risk of SPRA and observed consistent effects (C1s: OR 0.81, 95% CI 0.69–0.95, *P* = 1.1 × 10^−2^; C1r: OR 0.79, 95% CI 0.66–0.94, *P* = 9.9 × 10^−3^; Supplementary Figure S9).

**Figure 4:**
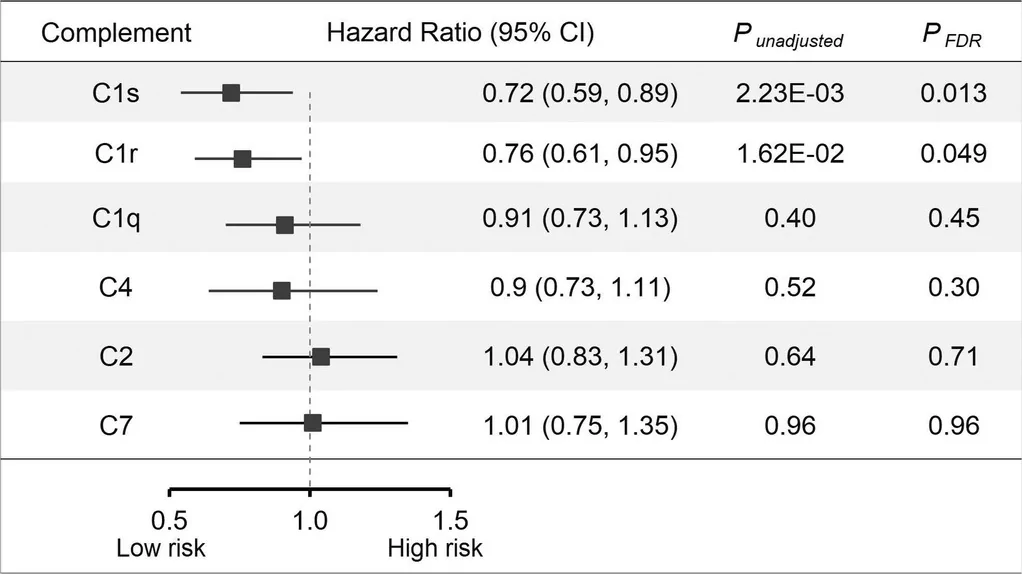
Effect modification of CHIP–SPRA association by genetically predicted complement levels. Forest plots show the interaction between CHIP and genetically predicted complement protein levels on incident SPRA risk from Cox models after correcting for biologic sex, smoking status, and five genetic principal components. *P* values are shown before and after FDR correction. CHIP, clonal hematopoiesis of indeterminate potential; CI, confidence interval; FDR, false discovery rate; SPRA, seropositive rheumatoid arthritis.

## DISCUSSION

Our three‐biobank meta‐analysis found that CHIP was associated with an increased risk of incident SPRA, driven primarily by an increase in *DNMT3A*‐CHIP–associated, late‐onset SPRA. Furthermore, participants with *TET2*‐CHIP and SPRA had an increased risk of all‐cause mortality compared to participants with SPRA alone. Effect modification analysis found genetically predicted levels of classical complement proteins C1r and C1s may modify the CHIP‐associated risk of SPRA. In UKB, autosomal mCA and mLOY were associated with an increased risk of SPRA.

The association between CHIP and incident SPRA with absence of association between CHIP and prevalent SPRA presents an apparent conflict. Straightforwardly, this observation may be the result of the reality that CHIP rarely occurs before middle age and RA generally onsets in older adulthood. However, results presented here suggest that differential survival may explain part of the enigma, with CHIP^+^ patients with SPRA less likely to survive to the date of enrollment. Intriguingly, a final possibility is that the treatments rendered for RA may cause CHIP clones to shrink or resolve, as has been observed experimentally in nonhuman primates for one RA treatment, tocilizumab, though this phenomenon has not been observed in humans.^43^

Throughout this study, all analyses relating to SNRA were null. Due to the low incidence of SNRA (Table 1), this study had 80% power to detect a CHIP‐associated hazard ratio for SNRA of 1.5, substantially larger than the 1.3 HR that could be detected with 80% power for SPRA. Furthermore, though CHIP has a well‐known association with all‐cause mortality that robustly replicated in the cohorts studied here (HR 1.35; *P* = 9.4 × 10^−17^; Supplementary Figure S8), there was no statistically significant CHIP–mortality association among people with prevalent SNRA. This negative result was most likely due to the study's ~10% power to detect a CHIP‐associated effect with hazard ratio 1.35 among the 2,542 people with prevalent SNRA rather than a CHIP‐mortality rescue phenomenon associated with SNRA. In summary, discordant results between SPRA and SNRA may reflect lack of power, biologic differences between SPRA and SNRA, or the difficulty of studying SNRA with a biobank approach due to the high susceptibility for misclassification.

Negative effect modification by genetically predicted levels of early classical complement proteins C1r and C1s suggests that the classical pathway may buffer, rather than amplify, the CHIP‐associated risk of SPRA. One explanation is that higher baseline C1r and C1s activity promotes more efficient opsonization and clearance of apoptotic and cellular debris, limiting dysregulated inflammatory signaling in the context of CHIP. These results motivate targeted mechanistic studies to test whether complement‐mediated clearance pathways attenuate CHIP‐associated risk for RA and other inflammatory diseases of aging. We note that circulating levels of complement components, particularly C4, are strongly influenced by gene copy number variation. Due to the lack of a C4 copy number genome‐wide association study, the genetic instrument approach applied here is not feasible to assess how copy number variation influences the CHIP‐associated risk for RA. Direct measurement of C4 copy number could be performed to more comprehensively define the role of the classical complement pathway in CHIP‐associated RA risk. The strong association between mLOY and incident SPRA is consistent with prior work on prevalent SPRA.^22^ Still, it was observed only in one biobank and merits replication in other biobanks. Similarly, the association between autosomal mCA and SPRA was statistically sound but merits replication. If these effects were found to be consistent across cohorts, mechanistic studies could begin to determine the pathways that relate these larger scale somatic genetic changes to RA.

The strengths of this study include its large sample size, considering >1 million research participants for inclusion and exclusion and ultimately studying 612,989. The large sample size permitted use of a phenotyping approach that prioritized specificity over sensitivity, as is critical for valid inference of uncommon outcomes. The phenotyping approach combined diagnosis codes, laboratory values, and medication prescriptions, yielding an estimated 99% specificity and overcoming a critical weakness of recent biobank‐based work on CH and RA in which researcher ascertained RA status strictly from billing codes.^18^, ^19^, ^25^, ^44^ This study also made use of the longitudinal follow‐up data in each biobank to conduct age‐scale survival analysis, suggesting that CH may *cause* RA, rather than vice versa.

This study also has notable limitations. First, because general‐purpose genetic sequencing was used to infer CHIP status, the ascertainment was neither sensitive nor specific.^24^ Imperfect CHIP calls increase the risk of false‐negative studies and attenuate the discernable risk but do not increase the risk of false‐positive associations.^24^ Furthermore, the size of a CHIP clone can have profound implications for health outcomes but cannot be estimated with general‐purpose sequencing.^13^, ^45^ To address these weaknesses will require dedicated CHIP sequencing in the baseline samples of a cohort that has been followed longitudinally.

Second, the heterogeneity of biobank designs might complicate direct comparison of results across cohorts. Although sequencing depths were similar, recruitment strategies and the timing of blood sample collection differed. These differences likely contributed to the higher baseline prevalence of CHIP observed in the hospital‐based BioVU cohort compared to the population‐based AoU or UKB cohorts. To address this heterogeneity and the resulting differences in baseline risk, we used survival analyses on the age scale. This approach accounts for age‐related risk nonparametrically and, importantly, satisfied the proportional hazards assumption across all biobanks, whereas the time‐on‐study scale did not.^46^

Third, because incident SPRA and SNRA are uncommon and our approach prioritized specificity over sensitivity, there were few events among participants with each kind of CH. A cohort in which more participants had CH or more participants developed RA would provide more powerful statistical inference. Relatedly, patients can move between health care systems in the United States, and electronic medical records may not always be accurately transferred with them. Therefore, some participants in BioVU and AoU who were adjudicated as incident cases may have truly been prevalent cases, misclassified due to missing medical records.

In conclusion, SPRA is a complex autoimmune disease with antigen‐driven clonal proliferation of lymphocytes as a core pathophysiologic process. This study suggests that the pro‐inflammatory, antigen‐independent clonal proliferation of hematopoietic stem cells such as CHIP and mCA also play a role in SPRA pathogenesis. The classical complement cascade may modify the role of CH in RA pathogenesis. Work into mechanisms by which *DNMT3A*‐CHIP, mCA, and mLOY drive the increased risk of SPRA merits attention as these insights might guide development of early interventions for patients with CHIP to avoid SPRA or precision interventions for patients with SPRA based on their CHIP status.

## AUTHOR CONTRIBUTIONS

All authors contributed to at least one of the following manuscript preparation roles: conceptualization AND/OR methodology, software, investigation, formal analysis, data curation, visualization, and validation AND drafting or reviewing/editing the final draft. As corresponding author, Dr Corty confirms that all authors have provided the final approval of the version to be published and takes responsibility for the affirmations regarding article submission (eg, not under consideration by another journal), the integrity of the data presented, and the statements regarding compliance with institutional review board/Declaration of Helsinki requirements.

## Supporting information


**Disclosure form**.

**Appendix S1:** Supplementary appendix

**Figure S1:** Gene distribution of clonal hematopoiesis driver mutations
**Figure S2:** Age‐related prevalence of CHIP across cohorts
**Figure S3:** Time‐to‐event association of CHIP with incident SPRA by cohort
**Figure S4:** Time‐to‐event association of CHIP with incident SNRA by cohort
**Figure S5:** CHIP driver gene distribution among SPRA and SNRA patients
**Figure S6:** CRP levels among SPRA patients with and without CHIP and osteoarthritis controls.
**Figure S7:** Association of clonal hematopoiesis with prevalent RA
**Figure S8:** Time‐to‐event association of CHIP with mortality by cohort
**Figure S9:** Associations of genetically predicted Complements with SPRA risk in the overall population and among CHIP carriers.

**Table S1:** Supplementary Tables
