# Supplementary material for: Association of Clonal Hematopoiesis With Incident, Late‐Onset, Seropositive Rheumatoid Arthritis
Source: Arthritis Rheumatol. 2026 Apr 16;78(10):2063–73. doi: 10.1002/art.70133 (PMC13619074; doi:10.1002/art.70133)
Supplement: Supplementary file 2 [file ART-78-2063-s001.docx]

# Supplementary Figures

## Figure S1. Gene distribution of clonal hematopoiesis driver mutations

Pie chart showing the distribution of clonal hematopoiesis driver genes. *DNMT3A*, *TET2*, and *ASXL1*  together account for over 75% of all detected CHIP mutations, indicating that these three genes dominate the CHIP mutational landscape.

## Figure S2. Age-related prevalence of CHIP across cohorts

(**A**) Overall prevalence of CHIP increased steadily with advancing age, exceeding 10% after age 70. (**B**) Age-stratified CHIP prevalence across the BioVU, *All of Us*, and UK Biobank cohorts, showing consistent age-dependent increases but higher absolute rates in BioVU.

## Figure S3. Time-to-event association of CHIP with incident SPRA by cohort

This forest plot shows the associations of overall CHIP and individual driver gene mutations (*DNMT3A, TET2, ASXL1*) with SPRA incidence in each cohort and after meta-analysis. The column “N” indicates the number of CH carriers included, and “Events” represents the number of RA cases among these CH carriers. Hazard ratios (HRs) with 95% confidence intervals quantify the effect sizes.

## Figure S4. Time-to-event association of CHIP with incident SNRA by cohort

This forest plot shows the associations of overall CHIP and individual driver gene mutations (*DNMT3A, TET2, ASXL1*) with SNRA incidence in each cohort and after meta-analysis. The column “N” indicates the number of CH carriers included, and “Events” represents the number of RA cases among these CH carriers. Hazard ratios (HRs) with 95% confidence intervals quantify the effect sizes.

## Figure S5. CHIP driver gene distribution among SPRA and SNRA patients

Panels A and B display the distribution of CHIP driver mutations among individuals who developed seropositive RA (SPRA; left) and seronegative RA (SNRA; right). Bars represent the number of carriers for each gene, stratified by exonic function (frameshift deletion, frameshift insertion, nonsynonymous SNV, splicing, and stop-gain mutations). The bottom panels show the variant allele frequency (VAF) distributions for each driver gene among patients with incident SPRA (left) and SNRA (right). Violin plots depict the range and density of clone sizes, with individual data points overlaid.

## Figure S6. CRP levels among SPRA patients with and without CHIP and osteoarthritis controls.

Violin and box plots show the distribution of log-transformed CRP levels in three groups: SPRA patients with CHIP (SPRA_CHIP), SPRA patients without CHIP (SPRA_nonCHIP), and osteoarthritis controls. Pairwise comparisons were performed using Wilcoxon rank-sum tests with continuity correction. Boxplots depict medians and interquartile ranges, and violins represent kernel density estimates.

## Figure S7. Association of clonal hematopoiesis with prevalent RA

This forest plot shows the associations of overall CHIP and individual driver gene mutations (*DNMT3A, TET2, ASXL1*) with SPRA (**A**) and SNRA (**B**) prevalence after meta-analysis, and the associations of mCA (autosomal mCA, mLOX, mLOY) with RA prevalence in UKB. Logistic regressions were performed to test the associations after adjusting the same covariates as mentioned in Methods. The column “N” indicates the number of CH carriers included, and “Events” represents the number of RA cases among these CH carriers. Odds ratios (ORs) with 95% confidence intervals quantify the effect sizes.

## Figure S8. Time-to-event association of CHIP with mortality by cohort

This forest plot shows the associations of overall CHIP and individual driver gene mutations (*DNMT3A, TET2, ASXL1*) with mortality in each cohort and after meta-analysis. The column “N” indicates the number of CH carriers included, and “Events” represents the number of mortality among these CH carriers. Hazard ratios (HRs) with 95% confidence intervals quantify the effect sizes.

## Figure S9. Associations of genetically predicted Complements with SPRA risk in the overall population and among CHIP carriers.

Forest plots show odds ratios and 95% confidence intervals for the associations between genetically predicted complement components and SPRA risk in all participants and when restricted to individuals carrying CHIP. Because genetically predicted complement levels reflect stable, lifelong genetic predisposition rather than time-varying exposure, their associations with SPRA risk in the overall population and among CHIP carriers were evaluated using logistic regression models. Odds ratios represent the change in SPRA risk per unit increase in genetically predicted complement level. While no significant associations were observed in the overall population, genetically predicted C1s, C1r, and C4b were associated with lower odds of SPRA among CHIP carriers.
