# Supplementary material for: Association of Clonal Hematopoiesis With Incident, Late‐Onset, Seropositive Rheumatoid Arthritis
Source: Arthritis Rheumatol. 2026 Apr 16;78(10):2063–73. doi: 10.1002/art.70133 (PMC13619074; doi:10.1002/art.70133)
Supplement: Supplementary file 3 [file ART-78-2063-s002.docx]

Supplementary appendix

Diagnosis codes

In the UKB, relevant codes were identified by searching for concepts that match the regular expression ‘(R|r)heumatoid’ but not ‘(J|j)uvenile’, yielding 130 codes, which were manually reviewed to ensure no inappropriate codes were captured. RA SNOMED codes were categorized as SPRA, SNRA, or RA NOS based on matching either “positive” (17), “negative” (6), or neither (107) (**Supplementary Table 2**). Diagnosis codes were evaluated with no consideration as to whether they were assigned inpatient vs. outpatient.

Two codes of a class (SPRA, SNRA, or RA NOS) were required to categorize the participant as that class. In cases where a participant was classified as both SPRA and SNRA, they were assigned to whichever class had a higher code count. In cases where a participant was assigned both a sero-specific class (SPRA or SNRA) and RA NOS, they were assigned to the sero-specific class.

Medications

For all three biobanks, DMARDs were identified by searching the Observational Medical Outcomes Partnership (OMOP) ingredient table for values that match the regular expression that combines the list of DMARD names, then using the concept_relationship and concept_ancestor tables to find all drug prescriptions in the biobank that contain those ingredients.

Laboratory values

We identified concepts that capture rheumatoid factor (RF) and anti-cyclic citrullinated peptide (CCP) by searching the concept table, subsetted to measurement concepts, and manually reviewing resulting concepts. For RF we searched for concepts that match ‘(R|r)heumatoid factor’. For CCP, we searched for concepts that match ‘citrullinated’ but not ‘Homo|Urine|vimentin|viral’.

Quantitative laboratory values that had an associated upper limit of normal (ULN) were adjudicated as positive or negative based on comparison with the ULN. Quantitative laboratory values in AOU and BioVU without defined ULN were adjudicated based on the ULN present in other measurements with the same units, with manual review to ensure at least 90% of results were negative and consistency with clinical experience of the authorship team in the practice of rheumatology in the United States. This process yielded a cutoff for RF of 15 IU/mL, for CCP in U/mL of 20 U/mL and for unitless CCP of 0.94. Quantitative lab values in UKB without defined ULN were adjudicated based on the same quantile analysis and a review of the UK’s National Health Services Pathology Lab publications, which yielded a cutoff of 20 IU/mL for RF and 7 U/mL for CCP. Laboratory values that were textual in nature were considered positive if they matched ‘pos’ or ‘>’, negative if they matched ‘neg’ or ‘<’, and N/A otherwise.

Case Definition:

Patients were considered to have SPRA if they met either of these criteria:

RA NOS by diagnosis codes + DMARD + seropositive by lab values

OR

SPRA by diagnosis codes + DMARD + (seropositive OR N/A by lab values)

Patients were considered to have SNRA if they met either of these criteria:

RA NOS by diagnosis codes + DMARD + seronegative by lab values

OR

SNRA by diagnosis codes + DMARD + (seronegative or N/A by lab values)

Clonal hematopoiesis of indeterminate potential detection

Putative CHIP variants were screened using the following criteria: total sequencing depth ≥ 20, alternate allele read depth count ≥ 3, representation in both sequencing directions, and statistical exclusion of germline heterozygosity.

Genetically predicted complement protein levels

We retrieved publicly available genetic effect estimates from the INTERVAL study, which applied Bayesian ridge regression to estimate genetic effects on SomaLogic protein measurements.^33^ Complement protein levels were estimated among participants of European ancestry in BioVU (N = 116,082) and UKB (N= 260,119). The single nucleotide variants and their weights are provided in **Supplementary Table 5**. To minimize the risk of spurious associations, we removed proteins where in-sample predictive performance was poor, requiring R^2^ ≥ 0.2 (**Supplementary Table 6**). The genetically predicted complement levels were calculated by summing the weighted genotypes of all selected variants using the following formula:

$$Genetically predicted complement= \sum_{i = 1}^{n} \beta i\cdot{SNP}_{i}$$

where βi represents the estimated weight (i.e., the natural logarithm of the odds ratio [OR]) of the i-th SNP, derived from the reference datasets, and SNPi is the genotype dose of each risk allele for that SNP. Genetically predicted complement levels were scaled to mean 0 and standard deviation 1 to facilitate interpretation of effect estimates.

Genetic principal components

Genetic principal components (PCs) were computed separately within each cohort to account for population structure and residual ancestry-related confounding. PCA was performed using autosomal, bi-allelic SNPs restricted to common variants (minor allele frequency >1%) after standard quality control. To minimize correlation among markers, SNPs were linkage disequilibrium (LD)–pruned using a sliding-window approach (e.g., 50-SNP window, step size 5 SNPs, r² < 0.1). Long-range LD regions were excluded prior to PCA. Principal components were calculated using standard PCA software (e.g., FlashPCA2 or PLINK 2.0), and eigenvectors were derived from the genotype correlation matrix within each cohort. The first five genetic principal components (PC1–PC5), which captured the major axes of genetic variation, were included as covariates in all regression and survival models to adjust for residual population stratification.
